# Substrate Heterogeneity as a Trigger for Species Diversity in Marine Benthic Assemblages

**DOI:** 10.3390/biology12060825

**Published:** 2023-06-06

**Authors:** Katharina Romoth, Alexander Darr, Svenja Papenmeier, Michael L. Zettler, Mayya Gogina

**Affiliations:** Leibniz Institute for Baltic Sea Research Warnemünde, Seestrasse 15, D-18119 Rostock, Germany; alexanderdarr@web.de (A.D.); svenja.papenmeier@io-warnemuende.de (S.P.); michael.zettler@io-warnemuende.de (M.L.Z.); mayya.gogina@io-warnemuende.de (M.G.)

**Keywords:** habitat complexity, macrozoobenthos, Baltic Sea, species richness, rare species

## Abstract

**Simple Summary:**

An increasing number of different habitats leads to an increasing number of species and has been considered a key driver for biodiversity. However, there is no common understanding on how to measure habitat diversity. In this study, we tested a newly proposed measure of substrate heterogeneity by classifying changes on the seafloor with underwater video imaging. This analysis showed that the presence of small patches of different soft sediment types was associated with elevated species richness and a higher rate of occurrence of rare species.

**Abstract:**

Many studies show that habitat complexity or habitat diversity plays a major role in biodiversity throughout different spatial scales: as structural heterogeneity increases, so does the number of available (micro-) habitats for the potential species inventory. The capability of housing species (even rare species) increases rapidly with increasing habitat heterogeneity. However, habitat complexity is not easy to measure in marine sublittoral sediments. In our study, we came up with a proposal to estimate sublittoral benthic habitat complexity using standard underwater video techniques. This tool was subsequently used to investigate the effect of habitat complexity on species richness in comparison to other environmental parameters in a marine protected area situated in the Fehmarn Belt, a narrow strait in the southwestern Baltic Sea. Our results show that species richness is significantly higher in heterogeneous substrates throughout all considered sediment types. Congruently, the presence of rare species increases with structural complexity. Our findings highlight the importance of the availability of microhabitats for benthic biodiversity as well as of the study area for regional ecosystem functioning.

## 1. Introduction

Species composition and species richness of faunal communities are well known to depend on different environmental factors with respect to the considered spatial scale [1]. For example, diversity and species richness of endobenthic macrofauna assemblages in the Baltic Sea are mainly influenced by salinity at regional scale of hundreds of kilometers (i.e., the entire sea or its southwestern part, stretching through several sub-basins [2,3]). By contrast, on a sub-regional scale of tens of kilometers, substrate characteristics and other factors, often masked by water depth, become more important [4,5]. However, many terrestrial, limnic, and marine studies show that habitat complexity, or habitat diversity, plays a major role in biodiversity throughout different spatial scales (e.g., [6,7,8]): as structural heterogeneity increases, so does the number of available (micro-) habitats for the potential species inventory [9]. The effect of the available number of habitats might not only be additive in the sense that the species inventory of the different habitats is added in a small area, but also that highly specialized species might even be endemic in these areas [10]. Furthermore, ecosystems that are more complex show higher levels of multiple ecosystem functions than ecosystems with low habitat diversity [11]. The capability of housing species (even rare species) increases rapidly with increasing habitat heterogeneity. On the other hand, common and rare species potentially play an important role in ecosystem functioning, either by offering novel contributions to functional diversity or via functional redundancy [12]. In addition, in natural or anthropogenic stress phases, communities with an extensive set of functional traits have a higher probability of surviving and contribute to the stabilization of the system [13]. 

However, habitat complexity is not easy to measure in marine sublittoral sediments. On larger scales, seafloor morphology is often used as a proxy to capture habitat heterogeneity [14]. On smaller scales, heterogeneity in sediment characteristics can be a key factor for determining species diversity (e.g., [5]). Nevertheless, sediment heterogeneity is often not captured in standardized sampling with a low number of replicates at individual stations. Hence, sediment composition in heterogeneous areas can be determined by significantly increasing the number of repetitions in physical sampling, leading to a huge amount of additional effort [15]. Another challenge arises in connection with the amount of sediment that is taken to analyze sediment characteristics. Taking a small sub-sample for sediment analysis often does not represent the full range of the present grain size span. This is why sediment and infauna samples are often taken separately to guarantee enough sample material for both analyses [16]. However, taking separate samples to estimate substrate heterogeneity may lead to potentially significant spatial mismatches between biogenic and geological data and may restrict the ability to cover the full range of available sediment structures. In addition, potentially important geogenic and biogenic structures such as boulders, pebbles, macrophyte meadows, and bivalve shells are often overlooked. An alternative, efficient way to estimate habitat complexity of a patch is through the use of underwater video that often accompanies the physical sampling [17]. 

In our study, we came up with a proposal to estimate sublittoral benthic habitat complexity using standard underwater video techniques. Data derived using this tool were subsequently used to investigate the effect of habitat complexity on species richness in comparison to other environmental parameters in an area with steep environmental gradients at a relatively small spatial scale of a few tens of kilometers.

## 2. Materials and Methods

### 2.1. Study Area

**Figure 1:**
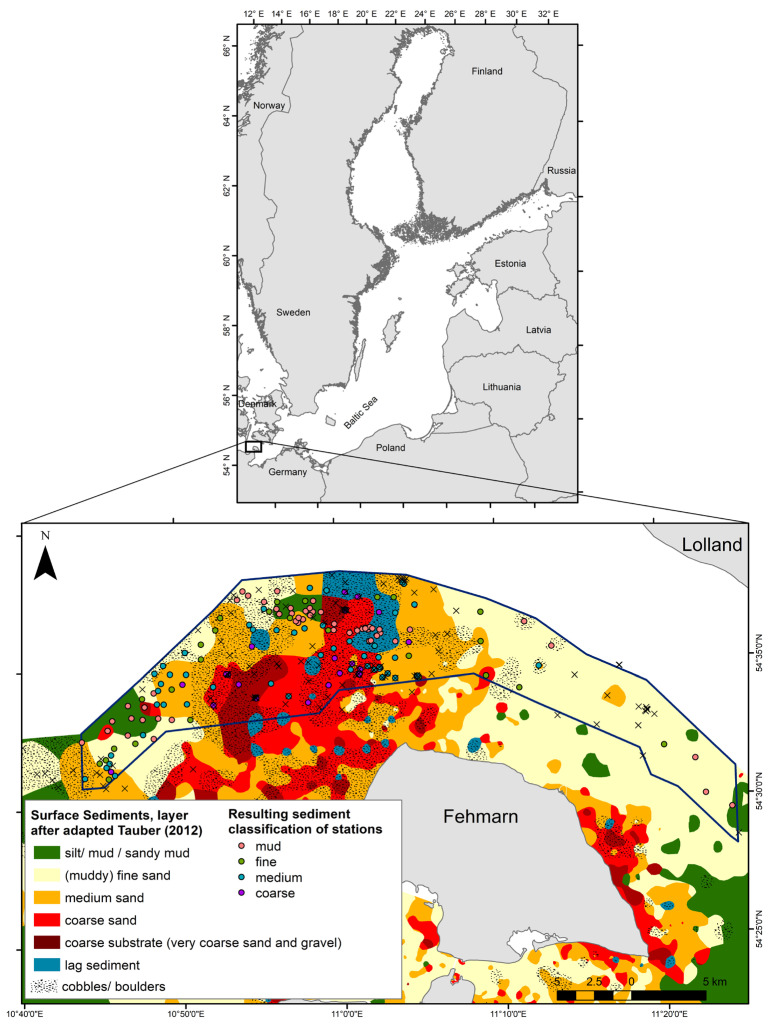
Map including the expected distribution of sediment types (after Tauber, 2012 [24]) and the location of sampling stations. Circles indicate 162 sampling locations (further referred to as stations) used for statistical analyses and the construction of general linear models, with circle colors referring to the results of the sediment type classification (crosses indicate samples excluded from the initial dataset (*n* = 355) due to the criteria listed in Section 2.2). The blue line indicates the border of the Natura 2000-site and the white area indicates unmapped seabed in Danish waters.

The Natura 2000-site “Fehmarn Belt” (EU-code DE 1332-301, hereafter referred to as marine protected area, MPA) is located in the southwestern Baltic Sea and covers an essential part of a narrow strait between the Danish island Lolland and the German island Fehmarn (Figure 1). It covers an area of 280 km² and is characterized by a steep depth gradient. The Fehmarn Belt is part of the Baltic transition zone that is influenced by the inflow of saline water from the Atlantic and the outflow of brackish water from the Baltic Proper [18,19]. More than two-thirds of the water volume exchanged between the North Atlantic and the Baltic Sea passes by the Belt Sea and, hence, through Fehmarn Belt [20]. 

Seafloor morphology and surface sediment structure in this area are formed out of glacial and postglacial processes. While the eastern and the southwestern parts of the MPA mainly consist of wide areas of muddy sand and sandy mud, the central part of the area is characterized by a high grain size heterogeneity [21]. This part of the study area consists of a large abrasion platform with the lowest water depths of 10 m cut by a deep valley (Vinds Grav channel) from east to west. The highest water depth here is around 40 m [22], filled with fine-grained deposits. Coarse lag deposits dominate the abrasion platform north of the incision. Boulders, pebbles, shell gravel, and sand of different grain size form a highly patchy mosaic of microhabitats. Sediment classes can change within meters. Similar deposits can be found south of the valley but with increasing distance, closer to the coast of Fehmarn, sand partly covers the lag deposits. A remarkable geological feature is a field of sand ribbons and drowned dunes of a height up to 2 m [23]. The sand dunes generally consist of medium-to-coarse sand with finer grain sizes dominating in higher depths below 18 m. In addition, accumulating *Arctica*-shells and floating kelp also increase habitat variability in this ribbon field.

### 2.2. Infauna and Sediment Data

Samples were collected during various projects at different spatial locations between 2012 and 2018. The locations (subsequently called stations) were selected in order to representatively cover the expected distribution of all major surface sediment types in the study area based on existing mapping, literature review, and the authors’ experience (see Figure 1). Although the sampled stations included both monitoring stations and randomly placed mapping stations, the applied sampling method remained the same over the seven years. At each station, at least one sample for infauna and one for the analysis of sediment properties was collected using a Van Veen grab (sampling area 0.1 m²), and one short video survey was conducted on the same day within a vicinity of 50–100 m. 

Infauna samples were sieved using a 1 mm mesh sieve and fixated using a 4% buffered formaldehyde seawater solution. In the lab, samples were sorted using a binocular with a 10-fold magnification, and individuals were identified to the lowest possible taxonomical level (mainly species level) and counted. Taxonomy followed the World Register of Marine Species (WoRMS). 

In contrast to the expected sediment types depicted in Figure 1, the final attribution of stations to the four sediment types was based on grain size distribution measurements using granulometric analyses. The considered sediment types were (i) muddy substrate (median grain size d50 < 63 µm), (ii) fine sand (d50 63–250 µm), (iii) medium sand (d50 250–500 µm), and (iv) coarse sand and gravel (d50 > 500 µm). The chosen number of classes was determined by the intention to have a sufficient number of stations within each class at the end and to adhere to the commonly accepted (coarse) sediment grain size classification. Sandy and gravelly sediments were dry-sieved automatically over a cascade of 10 sieves with differing mesh sizes ranging from 63 µm to 2 mm. Grain size distribution of muddy sediments was analyzed without chemical treatment by laser-diffraction particle size analyzer CILAS 1180L (3P Instruments GmbH & Co. KG, Odelzhausen, Germany). Parameters describing cumulative grain size distribution (namely median grain size, sorting, and skewness) were then calculated by using a skewed s-shape function, fitted to the cumulative grain size data with the least sum of squares method, applying a special fitting algorithm (the description is given in [25]). However, due to locally heterogeneous sediment conditions, additional information on sediment composition of the infauna sample was taken from the on-board optical sediment description. Samples were rejected from the analysis if a substantial mismatch occurred between the parameters of sediment sample and the on-board description of the sediment of infauna sample. 

Depending on the particular aim of the project, one or three replicates were taken per infauna sampling event. However, only one randomly selected infauna sample per location and sampling event was included in the analysis to avoid spatial dependencies. To eliminate the overwhelming effects from epibenthic communities, samples with hard substrate (boulders exceeding approximately 5 cm in diameter) or kelp were also excluded from the analysis before randomly selecting one sample per site. Overall, 162 stations (unique sampling events at location) with infauna and sediment information were finally included in the analysis (Figure 1).

Unattached sessile epibenthic specimens as well as specimens that were not identified to species level were excluded from the following analysis. In addition, oligochaetes were excluded, as they were identified to species level only in some of the campaigns. In contrast, a few genera were included if they were never identified to species level and if the genus was known to be represented by a single species in the regional dataset (e.g., *Autolytus*, *Phoronis*, *Edwardsia*). Additionally, the frequency was determined, which represented the percentage of stations at which a species occurred. Rare species received special attention in the following analysis and were here defined as species occurring at fewer than four stations, corresponding to a frequency of <2%, and at none of the stations present in the abundance exceeding 3 individuals per sample (0.1 m²). 

Video transects were taken using a towed system with a SeaViewer underwater camera. Until 2014, an analogous camera was used that was subsequently replaced by an HD camera of the same make. The video platform was equipped with additional light and towed over ground behind the floating vessel with the viewing direction ahead. The towing speed depended on the currents and wind speed and varied between 0.2 and 0.7 kn. The seabed was usually recorded for 5 minutes at approximately 0.5 m above the ground, depending on turbidity conditions. Only the first five minutes of the video were analyzed if the recording time exceeded this time span. Seafloor structures were categorized as follows: large boulders (hard substrates >50 cm), cobble/small boulders (hard substrates 5–50 cm), coarse gravel (2–5 cm), fine gravel (<2 cm), coarse or medium sand, fine sand, mud, bivalve shells (undestroyed or large pieces), and shell gravel. As the system was not equipped with laser pointers, no area calculation was possible. Consequently, the apparent occurrence of abiotic features was classified by estimation of the coverage using the following classes: absence of the feature, occasional occurrence (coverage <1% of the seafloor), frequent occurrence (1–10% coverage), dense occurrence (10–50%), and very dense occurrence (≥50%). For consistency in the video analysis and to avoid introducing observer-specific artefacts, the same person analyzed all the videos. As the video analysis only allows for a semi-quantitative approach, the substrate heterogeneity was described categorically. The four categories were defined as follows (see also Table 1 for schematic presentation):

- No heterogeneity (none): Other than the dominant (very dense) substrate class, at most one additional feature occurs occasionally;
- Low heterogeneity: Other than the dominant (very dense) substrate class, at most three additional features occur occasionally, or at most two additional features occur occasionally or frequently;
- Medium heterogeneity: Other than the dominant (very dense) substrate class, at most five additional features occur occasionally, or at most three additional features occur frequently of which one feature might occur densely;
- High heterogeneity: Any other combination, including at least four feature classes. Often, no single feature exceeds 50% coverage.

### 2.3. Analyses and Statistics

In this study, the species richness parameter was chosen to represent the species diversity. This metric is commonly used in studies addressing effects of habitat heterogeneity and complexity on biodiversity [26,27,28]. The Shannon–Wiener Index could alternatively be used [29,30], but equitability in distribution of species among a sample was outside the focus of this study.

All analyses were performed within the R environment [31]. Tests for normality in species richness were performed using a Shapiro–Wilk test [32]. Kruskal–Wallis and pairwise Mann–Whitney tests were used to initially evaluate the overall differences in species richness between the sediment types and substrate heterogeneity classes [33,34]. 

To compare diversity properties and account for possible sampling effort bias in estimating the expected number of observed species per sediment type, species-accumulation (rarefaction) curves were derived using the specaccum command of the R package vegan [35]. Default specaccum settings were used.

The dependency of species diversity on different environmental factors was tested using generalized linear models (GLM). GLM was chosen as the modelling method, as it was expected to have a higher power than linear models when analyzing count data [36]. The Shapiro–Wilk normality test suggested that species richness was not normally distributed. First, Poisson distribution was assumed for species richness (supported by the results of the Wilcoxon rank sum test) and correlations between numerical predictor variables were explored (see Supplementary Materials, Explanatory Text S1 for test results and Figure S3 for correlation graphs between numerical predictor variables). To reduce the complexity and find the best model, non-significant predictors were dropped, and backward selection based on the AIC information criterion [36] was carried out as the final step. However, the best-fitted Poisson model indicated overdispersion. To evaluate overdispersion, the DHARMa R package was used [37]. The variance was 3.8 times larger than the mean: plotted Pearson residuals considerably exceeded 1 (see Supplementary Materials, Explanatory Text S2). In order to address the detected overdispersion, we changed our distributional assumption to the negative binomial. To check if the distribution assumption could considerably influence our results, we also estimated the dispersion parameter within the model using the quasi-Poisson family. As there was no substantial difference in the interpretation, we focused on the outcome of the negative binomial model in the results, whereas the results of both dispersion-adjusted final models, side by side, are reported in the Supplementary Materials (Explanatory Text S2).

Overall, nine environmental parameters were tested in the initial model. The sediment variables loss on ignition, median grain size, skewness, and sorting were derived from the sediment analysis. Median grain size (in µm) indicated two outliers (values above 1500 µm): their influential effect was removed by transforming variable to phi units before entering the model [38]. Salinity and water depth were taken from measurements accompanying the sampling event. Slope and bathymetric position index (BPI) were derived from the bathymetry map by BSH [39], using the benthic terrain modeler extension (BTM, version 3.0) in ArcGIS [40]. Finally, substrate heterogeneity was estimated as described above and included as a 4-level categorical variable into the model (categories: none, low, medium, high). Sampling year and season (spring and summer) were included in GLM to test effects of temporal trends and seasonality.

Prior to entry into the model, numerical predictors were tested for collinearity using Spearman rank correlation (as mentioned above), and for the set including categorical predictors, the rule of Generalized Variance Inflation Factor GVIF (1/(2 × Df)) < 2.2 (as equivalent to simple variance inflation value VIF < 5) was applied. Values of GVIF suitable for categorical predictors were adjusted to make them comparable across different numbers of parameters, as recommended by Fox and Monette [41]. Potentially important environmental parameters, such as oxygen depletion or the portion of particular grain size fraction in the sediment, were excluded from the analysis after testing for variable collinearity.

In order to obtain more insights on where the differences captured by the final model came from, a post hoc test was carried out for between-subject factors and interactions. For post hoc test, the emmeans R package [42] was used with the default settings of Tukey method for comparing estimates.

## 3. Results

### 3.1. Overall Species Inventory

**Figure 2:**
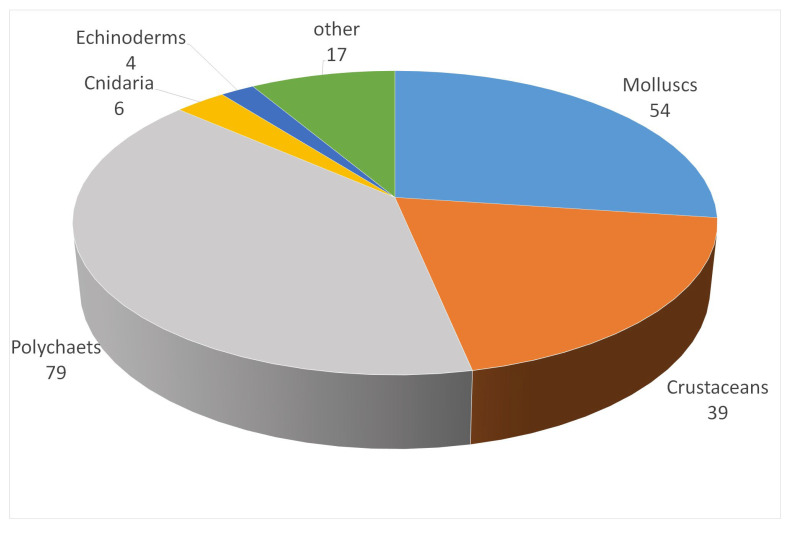
Composition of collected organisms. Polychaetes, molluscs, and crustaceans contributed most to species richness.

Overall, 199 species were identified, with polychaetes (79 species), molluscs (54), and crustaceans (39) being the main contributors to species richness (Figure 2). Few species were present throughout the area, with *Scoloplos armiger* (147 records, frequency 90.7%), *Kurtiella bidentata* (140, 86.4%), *Diastylis rathkei* (131, 80.9%), *Ophiura albida* (122, 75.3%), and *Abra alba* (122, 75.3%) being the most commonly occurring species. Overall, only 18 species were present in more than half of the stations. On the other hand, 25 species were identified in a singular sample and 48 species could be considered as rare in our dataset, following the definition given above (i.e., those occurring at frequency below 2% and with abundance at any station not exceeding 3 individuals per 0.1 m² sample). A complete list of species is provided in the Supplementary Materials—File S1. Of all the 199 species observed, 84 species were shared between all 4 sediment types considered, 9 were found only in mud, 2 were unique for fine sands, 14 for medium sands, and 15 for coarse sediments.

### 3.2. Species Richness in Different Sediment Types

**Figure 3:**
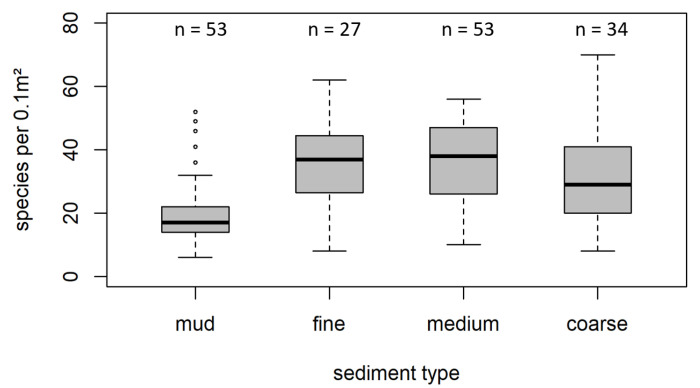
Boxplot showing species richness per grab and sediment type (mud, fine sand, medium sand, and coarse substrate). Boxes indicate the 25–75% interval, whiskers the 5–95% interval. Note that the black lines represent the median values that differ from the mean. Corresponding number of samples per category are given above each bar. Outliers are marked with circles.

Species richness varied between 6 and 70 species identified per 0.1 m², with a median of 27 species per sample. Median species richness per sediment type per sample varied between 17 taxa and 38 taxa per 0.1 m², with the lowest values in muddy substrate and the highest values in fine and medium sand (Figure 3). Although species richness in mud was significantly lower than in all other substrates (*p* < 0.001), no significant difference between the other sediment types were detected., Shapiro–Wilk tests for normality failed, indicating a non-normal distribution of species richness for all sediment types (Supplementary Materials—File S1).

**Figure 4:**
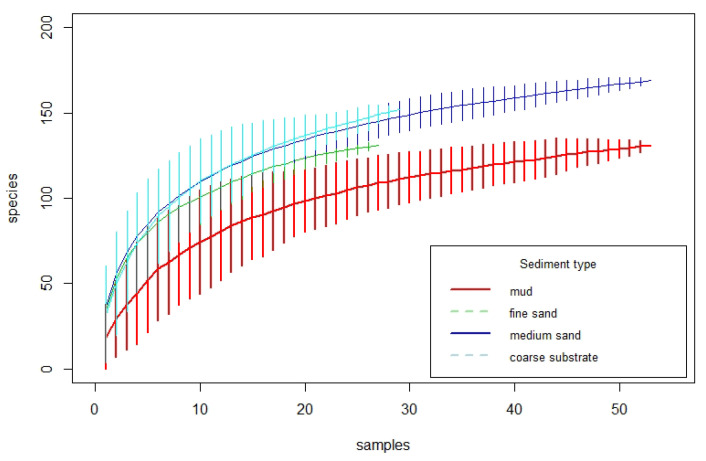
Species area curves for the four sediment types: mud (*n* = 53), fine sand (*n* = 27), medium sand (*n* = 53), and coarse substrate (*n* = 29), with vertical bars indicating the confidence interval at each step. A sample covers 0.1 m².

Results from the species area curves (rarefaction analysis) for different sediment types consistently showed the lowest species richness in muddy substrates (Figure 4). The course of the species area curves for fine sand flattened earlier than the course for medium sand and coarse substrates. At an area of 1 m² (10 samples), 72 ± 10 species were identified in muddy substrates, whereas species richness exceeded 100 m^−2^ in fine sand (104 ± 8), medium sand (109 ± 6), and coarse substrate (108 ± 11). In muddy substrates, a comparable number of species (105 ± 8) could only be found by aggregating 25 samples (corresponding to a cumulative sampled area of 2.5 m²). At this spatial scale, the species richness in fine sand (129 ± 2) was also significantly lower than in medium sand (142 ± 5) and coarse substrate (146 ± 4).

### 3.3. Testing the Relevance of Other Environmental Parameters

A negative test for normality indicated that other parameters in addition sediment type might influence the species richness of benthic communities in the investigation area. To explore the relative importance and explanatory power of both sediment type and substrate heterogeneity and to test for the influence of other parameters, a GLM was performed. 

We included the years of sampling treated as a continuous variable in the GLM, in order to evaluate the presence of any temporal trend. Stations were sampled either in spring (*n* = 36) or in summer (*n* = 126). To test seasonality, we also included the season and its interaction with the sediment type. Mud results, in particular, suggested a significantly higher mean number of species in spring. However, this seasonal difference must be treated with caution, as it could be caused by the lower number of spring samples and be an artefact of an admittedly unbalanced sampling design, especially as the summer observations with the highest values of species richness in mud were statistically treated as outliers (see boxplot in Supplementary Materials). The post hoc analysis results (Supplementary Materials, Tables S3–S5) provided more insights on the significant between-classes differences of this interaction term: pairwise comparison of individual classes revealed only significantly lower number of species in spring observed in coarse sediment type compared to summer values in fine and medium sand sediment types in our dataset. 

Median grain size (in phi units) had no significant effect on species richness when sediment type was included as a predictor and was dropped from the final model (the effect plot for this variable in the full model can be found in Supplementary Materials—File S1). The variable year was significant and had a negative estimate, suggesting some consistent reduction in species richness during the study period, particularly in “coarse” substrate and “fine” sand. Here, it is important to acknowledge the limits of this statistical inference due to possible temporal pseudoreplication. Sediment type and substrate heterogeneity both had significant effects on species richness. In particular, ‘none’ or ‘low’ heterogeneity showed the strongest linkage to a lower species number (Table 2 and Supplementary Materials—File S1). Common parameters describing seafloor topography (BPI and slope) where dropped from the final model for species richness.

**Figure 5:**
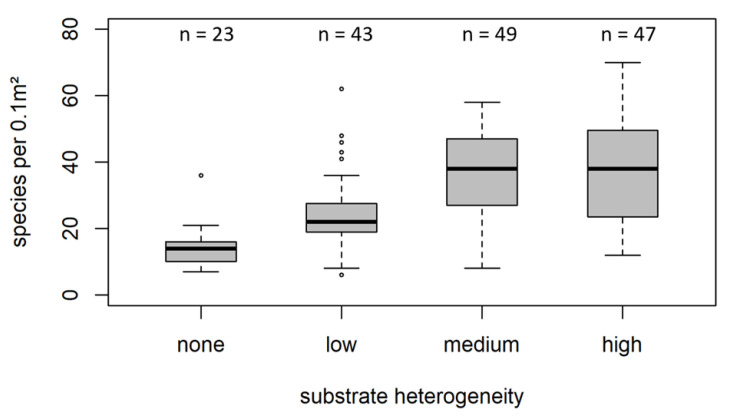
Boxplot showing species richness per grab and substrate heterogeneity class, as defined in Table 1. Boxes indicate the 25–75% interval, whiskers the 5–95% interval. Note that the black lines represent the median values that differ from the mean.

**Figure 6:**
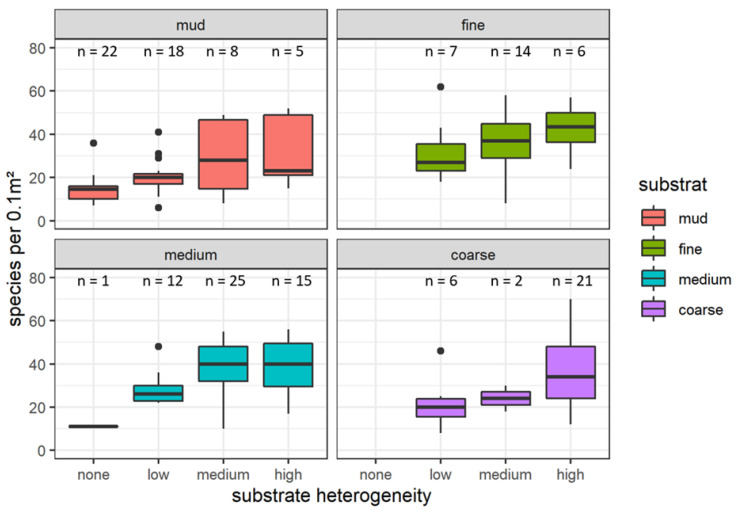
Boxplot showing species richness per grab separately for sediment types mud, fine sand (top line), medium sand, and coarse substrate (bottom) per substrate heterogeneity class. Boxes indicate the 25–75% interval, whiskers the 5–95% interval. Note that the black lines represent the median values that differ from the mean.

The influence of substrate heterogeneity on species richness was illustrated using a boxplot (Figure 5). In homogeneous substrates, species richness barely exceeded 20 species per 0.1 m² (median: 14 species per 0.1 m²). Species richness significantly increased by adding a few additional structural elements (substrate heterogeneity (GeoClass) ‘low’, median: 22 species per 0.1 m²) and even more at medium and higher substrate heterogeneity (38 per 0.1 m² for substrate heterogeneity (GeoClass) ‘medium’ and 37 for substrate heterogeneity (GeoClass) ‘high’). Looking separately at the four substrate classes described above revealed a similar pattern in all substrates (Figure 6). In all substrate classes, species richness was considerably lower in homogenous sediments or at low heterogeneity. However, due to the low number of samples in some combinations of substrate class and heterogeneity level, the significance of this pattern could not be verified. Results of the post hoc analyses (Supplementary Materials Figure S8) gives more detailed insights on species richness differences in independent categorical variables and interactions.

### 3.4. Occurrence of Rare Species

Overall, 80 records of 48 rare species were identified. Based on the number of records and the number of samples, the rate of rare species detected per sample was calculated. The rate successively increased from homogeneous substrates (0.22 rare species per sample) to 0.83 rare species per sample in very heterogeneous substrates, when summarized across all sediment types. The occurrence of rare species differed between the sediment classes. The lowest probability of finding a rare species was discovered in fine sand (0.11 rare species per sample), whereas statistically more than one rare species could be identified per sample in coarse substrates (1.17). Moreover, the probability of finding a rare species was highest in highly heterogeneous coarse substrates if heterogeneity and sediment classes were considered separately (Table 3).

## 4. Discussion

In this study, we tested a newly proposed measure of substrate heterogeneity. It was derived from the frequency of morphological structures on the seafloor recorded with underwater video, and it was attributed to seafloor heterogeneity at a spatial scale somewhat larger than that of a standard grab sample (roughly 40 m^2^ vs 0.1 m^2^ [17]). Our results suggest that the sediment information value from a grab sample can be limited, especially when the sediment in the grab is homogenous but comes from an overall heterogeneous surrounding.

The data used in this study were not based on experiments, but rather on various projects that have been carried out within the study area over several years. Such an approach often carries the risk of an unrepresentative distribution of stations with regard to the relevant environmental gradients. We acknowledge that the results should be interpreted with caution due to a possible temporal pseudoreplication. Also, in this study, the data points were not evenly distributed along the considered substrate gradient and the substrate heterogeneity classes. However, the fact that the combinations of sediment type and heterogeneity class were not evenly distributed in the data mainly originates from the genesis and the amount of sediment supplied [43]. The coarse sediments were relicts of glacial deposits and were granulometrically poorly sorted by nature. Permanent hydrodynamic forces, winnowing the fine fraction that accumulated in low energetic areas (e.g., depressions, stone shadow), reinforced the heterogeneity of lag sediment-dominated areas. The coverage of the southern abrasion platform with mobile sands and, thus, a homogenous sediment distribution was related to the availability of large amounts of reworked nearshore sediments [23]. In this study, we also included only one replicate per sampling event in the analysis. This absence of replication may increase uncertainty in our results and cause limited reliability, due to unaccounted patchiness and existing fine-scale variability in benthic fauna distribution, which should be kept in mind.

Additionally, the comparatively large period of seven years and the fact that the data originate from different seasons increases the included natural variability in the biological data and, consequently, the associated uncertainty in the results. As the Fehmarn Belt is situated at the entrance of the Baltic Sea, inhabiting communities are frequently influenced by protruding saline waters from the Kattegat and Skagerrak. These water masses potentially carry pelagic larvae and also adult specimens with them, temporarily complementing the autochthonous species inventory. However, as both homogeneous and heterogeneous sediments have been sampled throughout the full time span, it is unlikely that this had significant impact on the overall pattern of the results. 

In our study, we have focused on substrate characteristics and included comparatively few factors of water chemistry and physics that potentially may also affect species richness in the region. However, the included factors are known to be the most important for the distribution in the southwestern Baltic Sea and many other not included factors are known to be correlated with water depth; in particular, if the values describing them are derived from oceanographic models, this often remains the only option [44,45]. Additionally, other studies have shown that the available spatial resolution of such data (e.g., for drivers such as water currents or organic load) cannot act as a useful predictor on the considered scale of tens of kilometers [46]. Nevertheless, seasonal oxygen depletion mainly occurs in the deeper parts of the Fehmarn Belt and may (temporarily) reduce species richness in the predominantly homogenous muddy sediments. In addition, physical disturbance caused by anthropogenic activities, e.g., by demersal trawling, may have a negative impact on species richness (e.g., [47]). Bottom trawling mainly occurs on homogenous muddy and sandy sediments in the western and eastern parts of the study area [48], where species richness was detected to be comparably low. Nevertheless, due to limitations in our ability to adequately quantify the magnitude of this pressure in this area (c.f. [49]), its potential influence on species richness was not quantified here and needs to be addressed in future studies. 

The way to estimate habitat complexity varies considerably between different studies dealing with marine benthic habitats [9,14,50]. The diversity in approaches is partly related to the particular considered spatial scale and the availability of data to describe the habitat complexity. However, no common understanding on how to measure habitat diversity is available and, consequently, the studies are often hardly comparable. In our study, we used a simple classification scheme of structures and substrates detected using underwater video. As one person analyzed all videos and the same approach was applied to all records, the approach can be considered as standardized within the study. However, the selection of the included features and their classification remained subjective. One potentially crucial issue is the handling of the surrounding boulders inhabited by their own epibenthic-dominated communities [46]. As the target of the study was to detect the influence of substrate heterogeneity on soft sediment communities, we tried to avoid including samples randomly taken on boulders or patches of dense stones by excluding all stations with a corresponding description of the substrate. Nevertheless, the presence of small stones in the samples could not be ruled out. As small stones are often populated by species-poor communities [46,51] that correspond to those often found on large bivalve shells, which are in turn considered as structuring elements in soft sediments, it was unfeasible to a priori deselect all sessile species. Consequently, a few sessile and many characteristic accessory species of hard-substrate communities were found in the sample and significantly contributed to overall species richness, observed especially in heterogeneous substrates. Large shells from *Arctica islandica* are the dominating biogenic hard substrate and can be found throughout the whole study area. They provide settling space for small epibenthic species, such as barnacles, tunicates, and epibenthic bivalves, and shelter for mobile or tube-building species, e.g., of polychaetes genera *Harmothoe* and *Flabelligera*. Likewise, the surrounding geogenic hard substrates such as boulders and cobbles add to the species inventory of the soft-sediment communities. This happens either by detached biogenic material, such as floating algae or pieces of sponge colonies, carrying specimens that inhabit them or by mobile species. However, not only the presence of geogenic hard substrate and its epibenthic community, but also the presence of different soft sediment types on small patches significantly raised species richness. The positive effect of habitat heterogeneity on biodiversity has been demonstrated for both hard-bottom and soft-bottom in previous studies on benthic systems [8,52,53,54]. Explanations for the mechanisms behind this effect include a greater number of niches due to increased microhabitat availability and, associated with greater surface area, a higher productivity and sampling effect [7]. High substrate heterogeneity may form greater variation in space sizes, providing habitable space to organisms with a wider variety of body sizes, thereby leading to higher species richness [50,55]. Furthermore, Kovalenko et al. [7] argue that increasing habitat complexity may decouple trophic interactions and subsequently increase ecosystem stability. Overall, our findings are in line with the results of other studies from marine and brackish waters (e.g., [8,14]). It could be shown that the variety of ecological niches in the heterogeneous areas in MPA Fehmarn Belt not only raise local biodiversity but additionally, and more importantly, provide habitats for rare species that were not found in homogeneous sediments. The role of these rare species in ecosystem function and stability is still not fully understood, but most studies concordantly highlight their potential role in functional redundancy and, consequently, in securing ecosystem resilience [11,13]. Consequently, the integrity of the heterogeneous areas and the inhabiting communities in the Fehmarn Belt can be of special interest, not only for nature conservation, but also for ecosystem function of the whole area. 

## 5. Conclusions

To conclude, heterogeneous seabed forms structure habitat three-dimensionally, increase species richness, and buffer ecosystem functional diversity, thereby resisting fluctuating environmental factors. Areas with such a high multidimensional diversity are likely to be of outstanding importance in times of global overfishing, climate change, and exploration of offshore space and resources. The Fehmarn Belt is one these areas in the Baltic Sea, and its ecological development requires special attention to secure the future provision of related ecosystem services.

## Figures and Tables

**Table 1:** Classification of substrate heterogeneity using a number of substrate features identified in short video transects.

Substrate	Number of Features Occurring			
Heterogeneity Class	Occasionally	Frequently	Densely	Very Densely
	(≤1%)	(>1–10%)	(>10–50%)	(>50%)
none	≤1	0	0	1
low	≤3	0	0	1
or	≤2		0	1
medium	≤ 5	0	0	1
or	≤2		1	1
or	≤3			1
high	>5	0	0	1
or	>3			≤1

**Table 2:** Results of the final GLM obtained using negative binomial distribution to explore the dependency of response variable species diversity (number of species) on different environmental factors of interest, appearance of multiannual trends, and seasonal differences. Substrate heterogeneity classes are abbreviated as “GeoClass” in the table.

Model (AIC: 1246)					
	Estimate	Std. Error	t-Value	*p*	Significance
(Intercept)	307.1	93.2	3.30	0.001	***
Factor (GeoClass)—low	−0.37	0.09	−4.23	0.000	***
Factor (GeoClass)—medium	−0.05	0.09	−0.60	0.550	
Factor (GeoClass)—none	−0.62	0.15	−4.04	0.000	***
Depth	−0.03	0.01	−2.69	0.007	**
Salinity	0.04	0.01	2.83	0.005	**
Year	−0.15	0.05	−3.26	0.001	**
Factor (sediment)—fine	−144.7	174.5	−0.83	0.407	
Factor (sediment)—medium	−325.2	120.0	−2.71	0.007	**
Factor (sediment)—mud	−374.7	139.6	−2.68	0.007	**
Factor (Season)—summer	0.29	0.17	1.74	0.082	.
Year: (sediment)—fine	0.07	0.09	0.83	0.405	
Year: (sediment)—medium	0.16	0.06	2.71	0.007	**
Year: (sediment)—mud	0.19	0.07	2.69	0.007	**
Summer: (sediment)—fine	−0.26	0.28	−0.92	0.356	
Summer: (sediment)—medium	−0.15	0.22	−0.67	0.502	
Summer: (sediment)—mud	−0.55	0.23	−2.44	0.015	*

* Significance codes: *** *p* < 0.001; ** *p* < 0.01; * *p* < 0.05; *p* < 0.1; Null deviance: 322.5 on 161 degrees of freedom; Residual deviance: 165.9 on 145 degrees of freedom.

**Table 3:** Mean number of rare species per sample in different combinations of sediment classes and substrate heterogeneity. Numbers in brackets indicate the number of samples per combination. NA: combination not present.

Substrate Heterogeneity	Mud	Fine Sand	Medium Sand	Coarse Substrate	Overall
None	0.23 (22)	NA	0 (1)	NA	0.22 (23)
Low	0.11 (18)	0 (7)	0.42 (12)	0.83 (6)	0.28 (43)
Medium	0.25 (8)	0.21 (14)	0.72 (25)	0.50 (2)	0.49 (49)
High	0.60 (5)	0 (6)	0.53 (15)	1.33 (21)	0.83 (47)
Overall	0.23 (53)	0.11 (27)	0.58 (53)	1.17 (29)	0.49 (162)

## Data Availability

The data presented in this study are available on request from the corresponding author. The infauna data are going to be available via an online portal of the Federal Agency for Nature Conservation at the end of 2023 (contact: kathrin.heinicke@bfn.de).

## Supplementary Materials

The following supporting information can be downloaded at https://www.mdpi.com/article/10.3390/biology12060825/s1: Figure S1: Description of the occurring substrates at the study site; Explanatory Text S1: Testing the suitability of Poisson distribution; Figure S2: Poisson distribution; Figure S3: Checking predictors for collinearity; Table S1: Generalized Variance Inflation Factors; Figure S4: Effect plots for each predictor in the field model; Explanatory Text S2: Dispersion analysis and evaluation of how much the coefficient estimations are affected by overdispersion; Figure S5: Plot of estimated variance against the mean (Pearson residuals) for the best fitted Poisson model; Figure S6: DHARMa nonparametric dispersion test via sd of residuals fitted vs. simulated for the best fitted Poisson model: (dispersion = 3.8552, *p*-value < 0.0001) and plots of scaled residuals; Table S2: Results of GLM using the *Quasipoisson* family and alternatively used Negative Binomial instead of the Poisson model; Figure S7: DHARMa nonparametric dispersion test via sd of residuals fitted vs. simulated for negative binomial model (dispersion = 0.88992, *p*-value = 0.424) and plots of scaled residuals; Figure S8: Effect plots for each predictor in the final negative binomial model remained very similar; Tables S3–S5: Results of post-hoc tests for the final negative binomial GLM model. Upper triangle: *p* values adjust = “tukey”; diagonal: [Estimates] (emmean); lower triangle: Comparisons (estimate) earlier vs. later; Figure S9: Boxplot comparing the number of species in spring and summer; Figure S10: Positioning of stations sampled in mud; Figure S11: GLM results and effect plots for each predictor in the full model with the two influential points (outliers); Figure S12: Effect plots for each predictor in the full model with median grain size transformed in phi units; Table S6: List of species.
