# Supplementary material for: Substrate Heterogeneity as a Trigger for Species Diversity in Marine Benthic Assemblages
Source: Biology (Basel). 2023 Jun 6;12(6):825. doi: 10.3390/biology12060825 (PMC10295182; doi:10.3390/biology12060825)
Supplement: Supplementary file 1 [file biology-12-00825-s001.zip › biology-2333303-supplementary.pdf]

Supplementary Material File S1

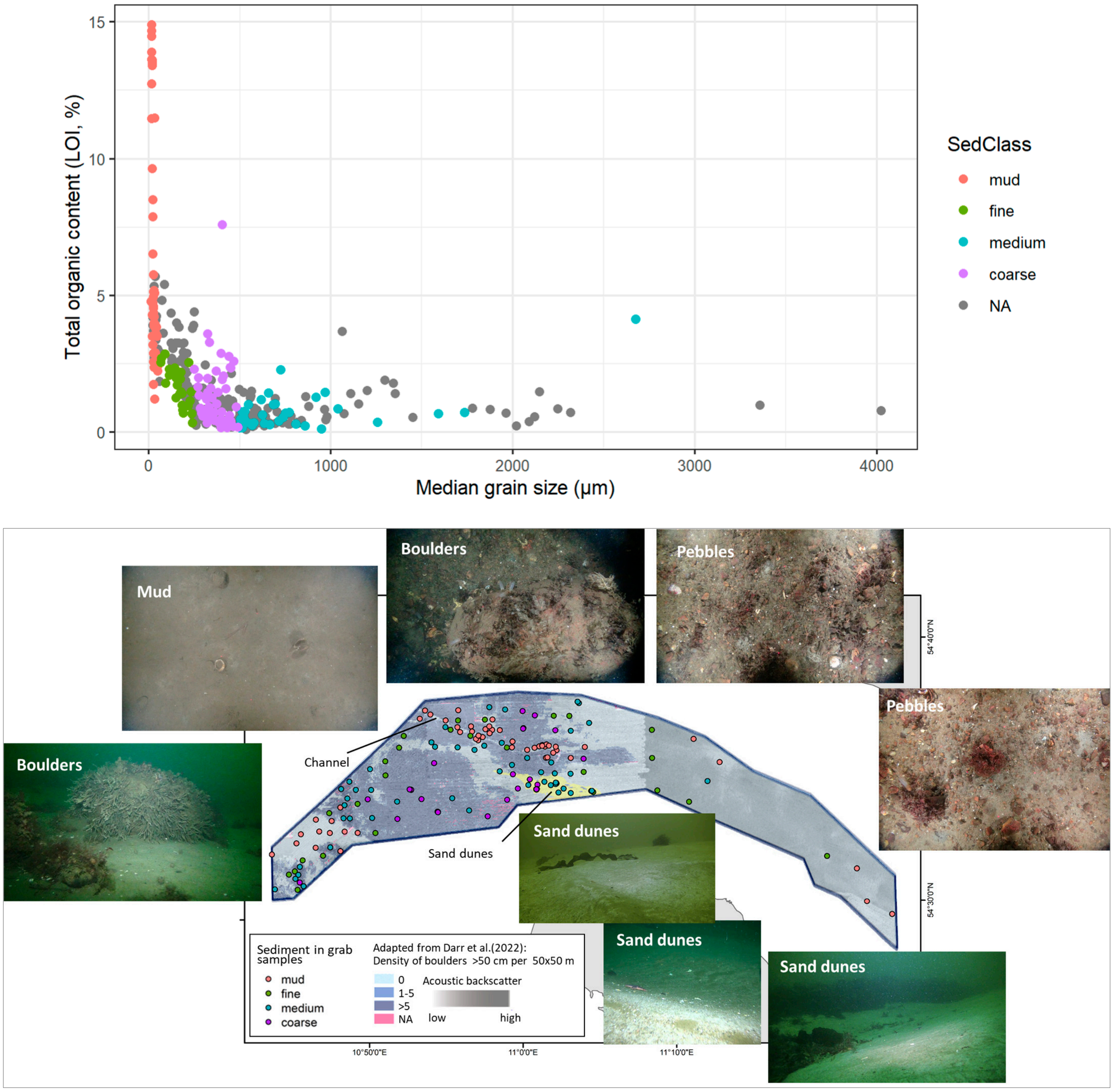

Figure S1. Description of the occurring substrates at the study site. Sediment median grain size based on granulomerty analysis (for the resulting assignment to one of 4 sediment type classes considered in this study refer to Figure 1 in the main text). The thresholds used to separate between the sediment types were (i) muddy substrate (median grain size d50 < 63µm), (ii) fine sand (d50 63-250µm), (iii) medium sand (d50 250–500µm) and (iv) coarse sand and gravel (d50> 500µm). NA indicates stations excluded from the analysis if the assignment to sediment type based on median grain size showed substantial mismatch with on-board optical description of sediment in the grab(s) collected for infauna. Values of median grain size are plotted against the total organic content estimated from the sediment sample using loss on ignition (LOI). Map with exemplary figures provides an overview with backscatter mosaic and boulder density layers adapted from Darr et al. (2022), location of sand dunes (in yellow), channel (visible from backscatter) and few photos to provide reader with the impression on mud habitat, sand dunes, pebbles and boulders around the study area.

Explanatory Text S1: Testing the suitability of Poisson distribution.

We have tested if the number of species (Nsp) follows normal or Poisson distribution using Shapiro-Wilk normality test. If the significance value of the Shapiro-Wilk test is greater than 0.05, normal distribution can be assumed for the data. If it is below 0.05, the data significantly deviate from a normal distribution. Number of species (Nsp) do not follow normal distribution:

Shapiro-Wilk normality test: W = 0.95837, p-value = 0.00009

Wilcoxon rank sum test with continuity correction comparing generated data with Poisson distribution and the same parameter $\lambda = 30.4$, same as mean species number in our data set:
W = 12338, p-value = 0.3521
At the 5% significance level, the null hypothesis that two samples come from the same distribution cannot be rejected, and we can assume our variable of interest to the Poisson distribution.

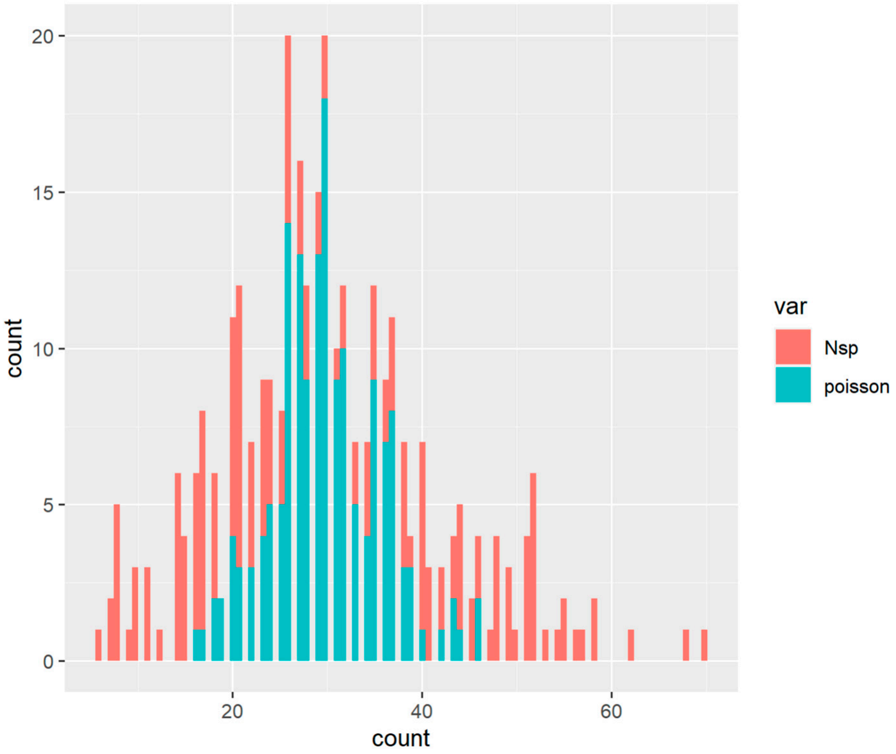

Figure S2. Poisson distribution.

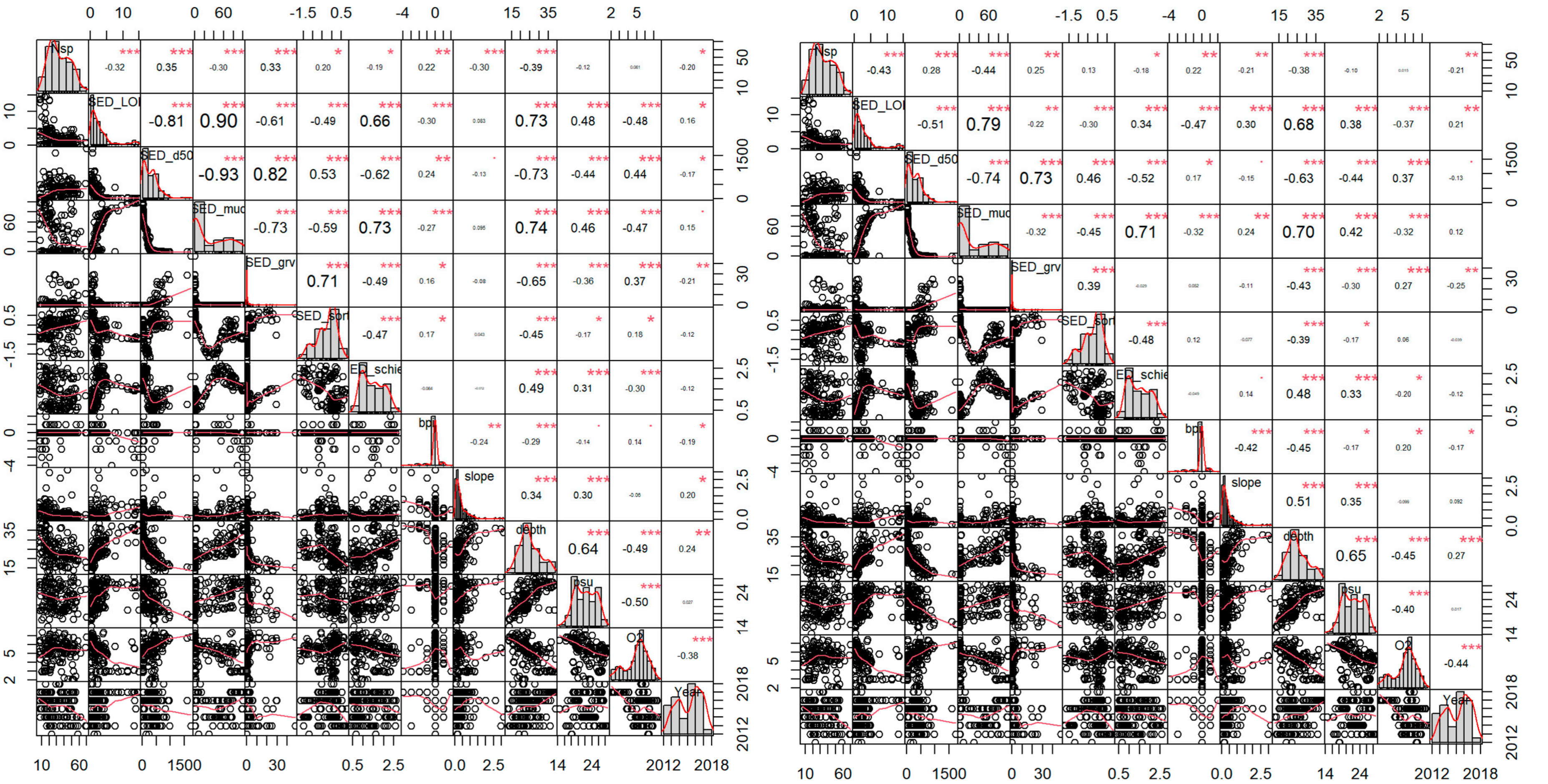

Figure S3. Checking predictors for collinearity. Left plot: Spearmann; right plot: Pearson.

Table S1. Generalized Variance Inflation Factors.

	GVIF	Df	GVIF^(1/(2*Df))
**Year**	1.707964	1	1.306891
**sediment**	75.07162	3	2.0539
**GeoClass**	3.50756	3	1.232634
**d50phi**	21.12618	1	4.596322
**SED_schief**	3.120841	1	1.76659
**SED_sort**	3.676853	1	1.917512
**depth**	4.70568	1	2.169258
**psu**	2.064483	1	1.436831
**bpi**	1.517588	1	1.231904
**slope**	1.490563	1	1.220886
**SED_LOI**	3.30874	1	1.818994
**Season**	1.623469	1	1.274154

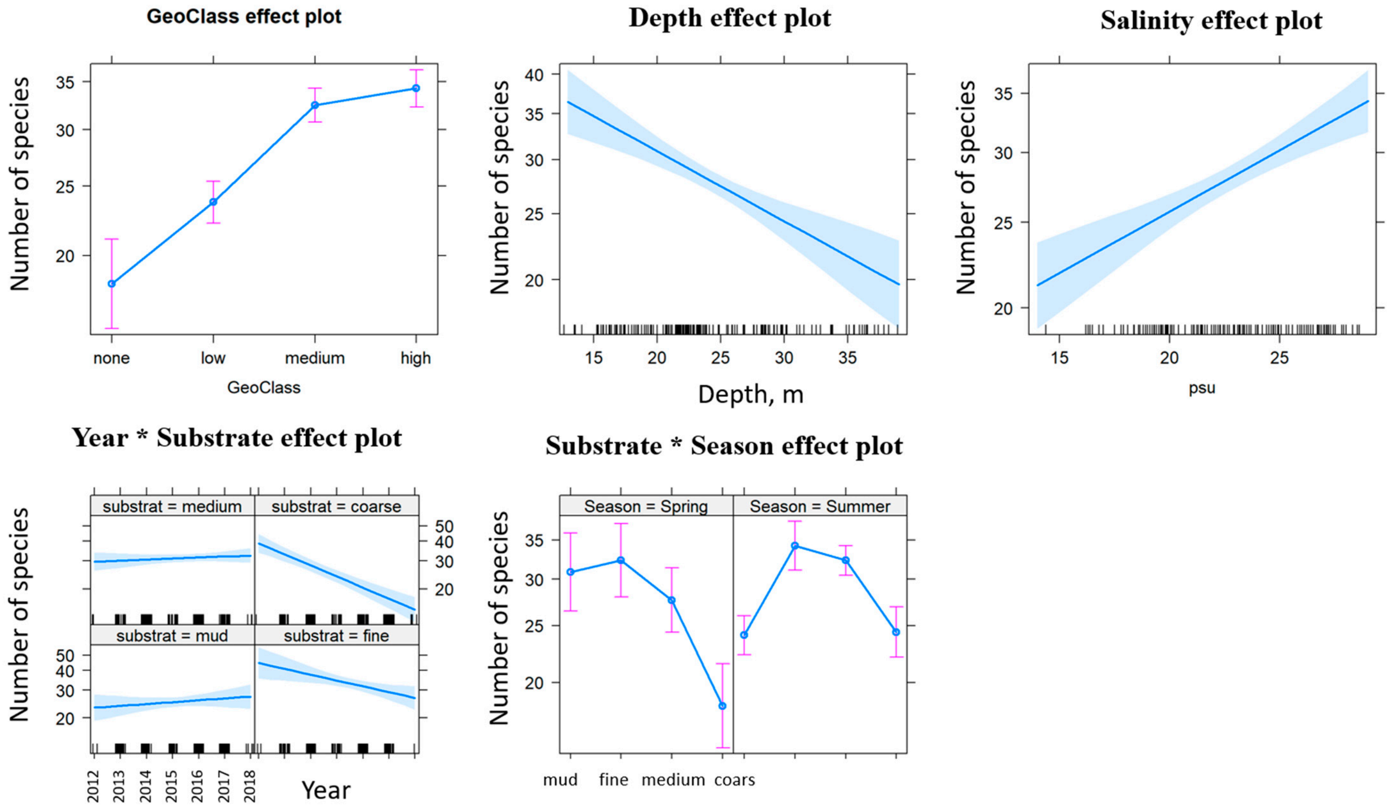

Figure S4. Effect plots for each predictor in the Poisson model (overdispersed).

Explanatory Text S2: Dispersion analysis and evaluation of how much the coefficient estimations are affected by overdispersion.

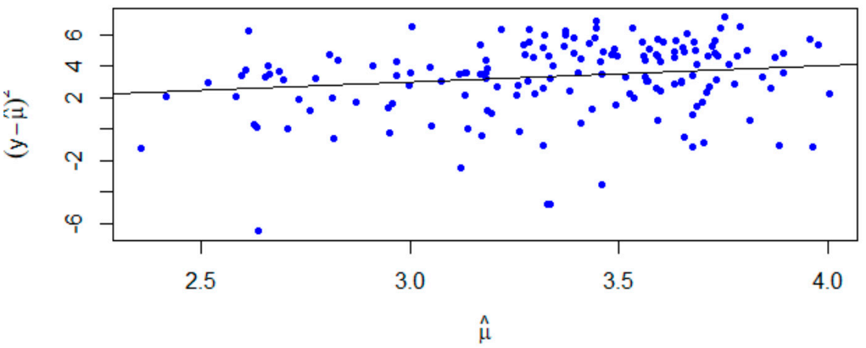

Figure S5. Plot of estimated variance against the mean (Pearson residuals) for the best fitted Poisson model.

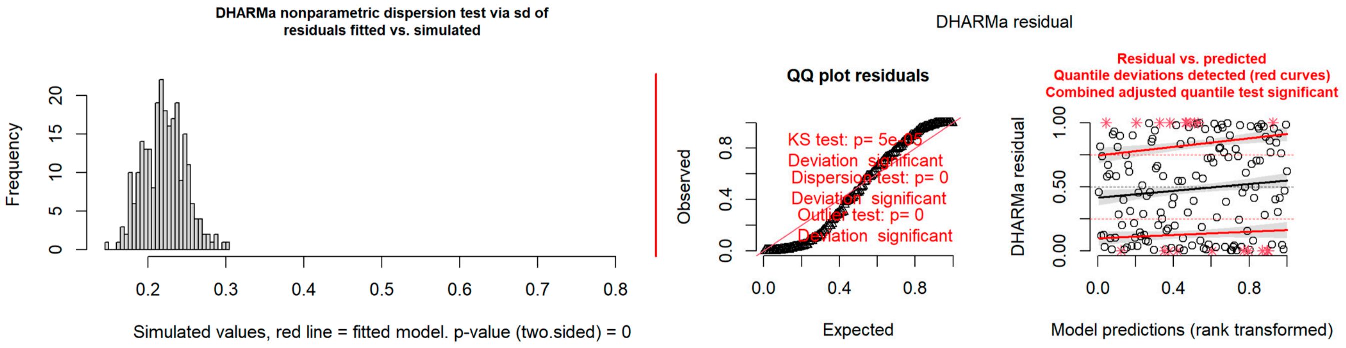

Figure S6. DHARMa nonparametric dispersion test via sd of residuals fitted vs. simulated for the best fitted Poisson model: (dispersion = 3.8552, p-value < 0.0001) and plots of scaled residuals.

To adjust for overdispersion we estimated the dispersion parameter within the model using the ***Quasipoisson*** family and alternatively used **Negative Binomial** instead of the Poisson model. Both model results are summarized below:

Table S2. Results of GLM using the *Quasipoisson* family and alternatively used Negative Binomial instead of the Poisson model.

	using quasi-family to estimate the dispersion					assuming negative binomial distribution				
	Estimate	Std.Error	t	value	Pr(>|t|)	Estimate	Std.Error	t	value	Pr(>|t|)
(Intercept)	326.7	93.0	3.51	0.001	***	307.1	93.2	3.30	0.001	***
Factor (GeoClass) – low	-0.37	0.09	-3.97	0.000	***	-0.37	0.09	-4.23	0.000	***
Factor (GeoClass) – medium	-0.05	0.08	-0.63	0.528		-0.05	0.09	-0.60	0.550	
Factor (GeoClass) – none	-0.63	0.18	-3.58	0.000	***	-0.62	0.15	-4.04	0.000	***
Depth	-0.02	0.01	-2.37	0.019	*	-0.03	0.01	-2.69	0.007	**
Salinity	0.03	0.01	2.52	0.013	*	0.04	0.01	2.83	0.005	**
Year	-0.16	0.05	-3.48	0.001	***	-0.15	0.05	-3.26	0.001	**
Factor (sediment) – fine	-151.8	161.5	-0.94	0.349		-144.7	174.5	-0.83	0.407	
Factor (sediment) – medium	-353.8	117.2	-3.02	0.003	**	-325.2	120.0	-2.71	0.007	**
Factor (sediment) – mud	-377.2	150.6	-2.51	0.013	*	-374.7	139.6	-2.68	0.007	**
Factor (Season) - Summer	0.29	0.18	1.65	0.102		0.29	0.17	1.74	0.082	.
Year: (sediment) – fine	0.08	0.08	0.94	0.347		0.07	0.09	0.83	0.405	
Year: (sediment) – medium	0.18	0.06	3.02	0.003	**	0.16	0.06	2.71	0.007	**
Year: (sediment) – mud	0.19	0.07	2.51	0.013	*	0.19	0.07	2.69	0.007	**
Summer: (sediment) – fine	-0.23	0.27	-0.87	0.387		-0.26	0.28	-0.92	0.356	
Summer: (sediment) – medium	-0.13	0.23	-0.59	0.559		-0.15	0.22	-0.67	0.502	
Summer: (sediment) – mud	-0.54	0.25	-2.18	0.031	*	-0.55	0.23	-2.44	0.015	*
	(Dispersion parameter for quasipoisson					(Dispersion parameter for Negative Binomial (10.25)				
	family taken to be 4.22)					family taken to be 1)				
	Null deviance: 1197.9 on 161 degrees of freedom					Null deviance: 322.5 on 161 degrees of freedom				
	Residual deviance: 613.2 on 145 degrees of freedom					Residual deviance: 165.9 on 145 degrees of freedom				
	Number of Fisher Scoring iterations: 4					AIC: 1245.9 Number of Fisher Scoring iterations: 1				
						Theta: 10.26				
						Std. Err.: 1.57				
						2 x log-likelihood: -1209.9				

Most of predictors' coefficients remained significant regardless of assuming Quasipoisson or negative binomial distribution, though some lost in significance level comparing to GLM model fitted using Poisson distribution. The exception was factor Season that became insignificant outside of the interaction with mud in case of both adjusted models. This caused little change in the interpretation of the model.

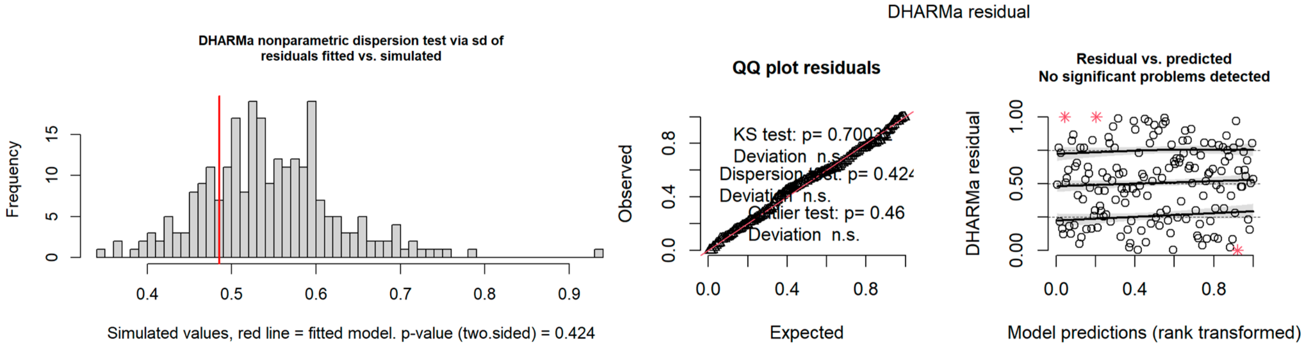

Figure S7. DHARMa nonparametric dispersion test via sd of residuals fitted vs. simulated **for negative binomial model** (dispersion = 0.88992, p-value = 0.424) and plots of scaled residuals.

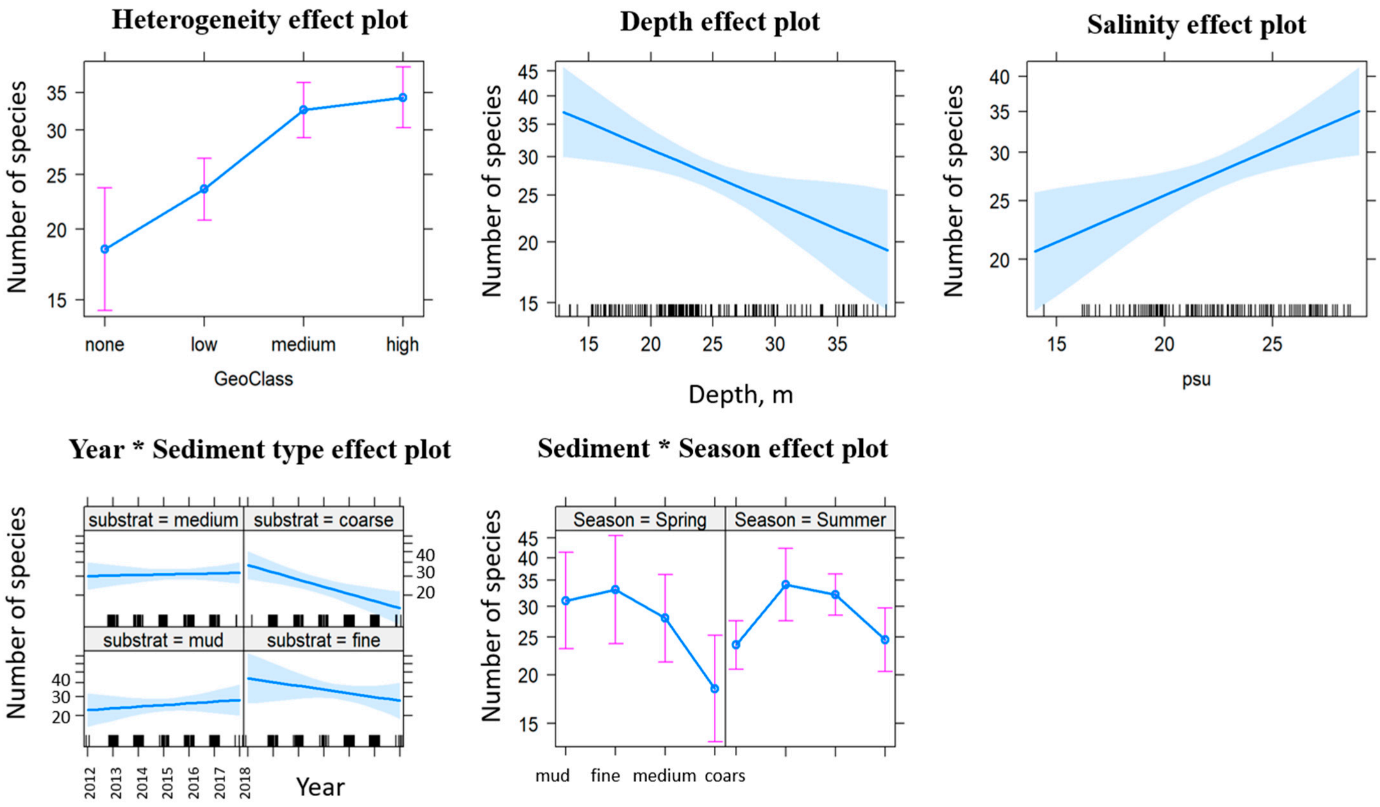

Figure S8: Effect plots for each predictor in the **final negative binomial model** remained very similar.

Results of post-hoc tests for the final negative binomial GLM model. Upper triangle: P values adjust = "tukey"; diagonal: [Estimates] (emmean); lower triangle: Comparisons (estimate) earlier vs. later.

Table S3. Row and column labels: GeoClass

	none	low	medium	high
none	[2.90]	0.360	**0.000**	**0.000**
low	-0.246	[3.15]	**0.001**	**0.000**
medium	**-0.568**	**-0.323**	[3.47]	0.933
high	**-0.620**	**-0.374**	-0.052	[3.52]

Table S4. Row and column labels: Season:sediment – note that pairwise comparison of individual classes revealed only significantly lower number of species in spring observed in coarse sediment type compared to summer values in fine and medium sand sediment types in our data set.

	Spring-mud	Summer-mud	Spring-fine	Summer-fine	Spring-medium	Summer-medium	Spring-coarse	Summer-coarse
Spring-mud	[3.38]	0.722	1.000	1.000	1.000	1.000	0.251	0.915
Summer-mud	0.263	[3.11]	0.595	0.142	0.971	0.073	0.851	1.000
Spring-fine	-0.064	-0.327	[3.44]	1.000	0.992	1.000	0.158	0.767
Summer-fine	-0.095	-0.358	-0.032	[3.47]	0.940	1.000	**0.032**	0.322
Spring-medium	0.105	-0.158	0.169	0.200	[3.27]	0.982	0.438	0.992
Summer-medium	-0.035	-0.298	0.028	0.060	-0.140	[3.41]	**0.022**	0.211
Spring-coarse	0.522	0.259	0.586	**0.617**	0.417	**0.557**	[2.86]	0.661
Summer-coarse	0.232	-0.031	0.296	0.328	0.127	0.268	-0.290	[3.14]

Table S5. Row and column labels: GeoClass:sediment

	none-mud	low-mud	medium-mud	high-mud	none-fine	low-fine	medium-fine	high-fine	none-medium	low-medium	medium-medium	high-medium	none-coarse	low-coarse	medium-coarse	high-coarse
none-mud	[2.89]	0.963	**0.007**	**0.005**	0.915	0.312	**<.0001**	**<.0001**	1.000	0.835	**0.003**	**0.002**	0.938	1.000	0.940	0.803
low-mud	-0.246	[3.13]	**0.016**	**0.003**	1.000	0.915	0.008	0.002	1.000	1.000	0.123	0.038	0.659	0.938	1.000	1.000
medium-mud	**-0.568**	**-0.323**	[3.46]	1.000	0.952	1.000	0.915	**0.919**	0.633	0.985	1.000	1.000	**0.014**	0.057	0.938	0.997
high-mud	**-0.620**	**-0.374**	-0.052	[3.51]	0.891	1.000	0.999	0.915	0.520	0.923	1.000	1.000	**0.017**	0.052	0.956	0.938
none-fine	-0.211	0.035	0.357	0.409	[3.10]	0.963	**0.007**	**0.005**	0.999	1.000	0.487	0.348	**0.033**	1.000	1.000	1.000
low-fine	-0.457	-0.211	0.112	0.163	-0.246	[3.34]	**0.016**	**0.003**	0.804	0.999	0.978	0.857	0.019	0.033	1.000	1.000
medium-fine	**-0.779**	-0.534	-0.211	-0.159	**-0.568**	**-0.323**	[3.67]	1.000	**0.006**	0.080	0.999	1.000	**<.0001**	**<.0001**	**0.033**	0.211
high-fine	**-0.831**	-0.585	**-0.263**	-0.211	**-0.620**	**-0.374**	-0.052	[3.72]	**0.005**	**0.035**	0.998	0.999	**<.0001**	**<.0001**	0.166	**0.033**
none-medium	-0.097	0.149	0.472	0.523	0.114	0.360	**0.683**	**0.734**	[2.98]	0.963	**0.007**	**0.005**	0.210	1.000	0.999	0.988
low-medium	-0.342	-0.097	0.226	0.278	-0.131	0.114	0.437	**0.489**	-0.246	[3.23]	**0.016**	**0.003**	0.119	0.210	1.000	1.000
medium-medium	**-0.665**	-0.420	-0.097	-0.045	-0.454	-0.209	0.114	0.166	**-0.568**	**-0.323**	[3.55]	1.000	**<.0001**	**0.000**	0.210	0.700
high-medium	**-0.717**	-0.471	-0.148	-0.097	-0.506	-0.260	0.063	0.114	**-0.620**	**-0.374**	-0.052	[3.60]	**<.0001**	**0.000**	0.482	0.210
none-coarse	0.246	0.491	**0.814**	**0.866**	**0.457**	0.702	**1.025**	**1.077**	0.342	0.588	**0.911**	**0.962**	[2.64]	0.963	**0.007**	**0.005**
low-coarse	0.000	0.246	0.569	0.620	0.211	0.457	**0.779**	**0.831**	0.097	0.342	**0.665**	**0.717**	-0.246	[2.89]	**0.016**	**0.003**
medium-coarse	-0.323	-0.077	0.246	0.297	-0.112	0.134	**0.457**	0.508	-0.226	0.020	0.342	0.394	**-0.568**	**-0.323**	[3.21]	1.000
high-coarse	-0.374	-0.129	0.194	0.246	-0.163	0.082	0.405	**0.457**	-0.278	-0.032	0.291	0.342	**-0.620**	**-0.374**	-0.052	[3.26]

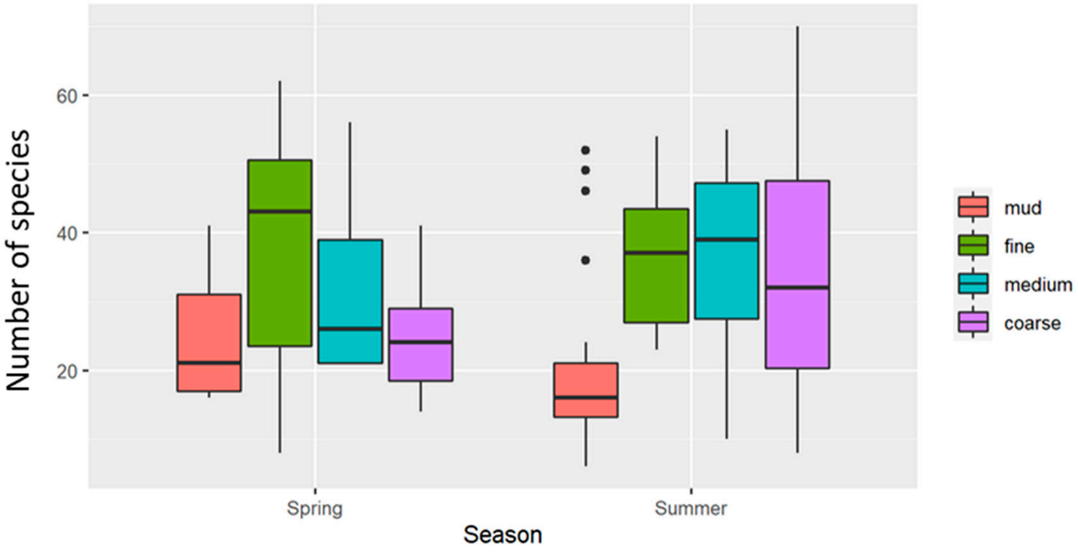

Figure S9. Boxplot comparing the number of species in spring and summer.

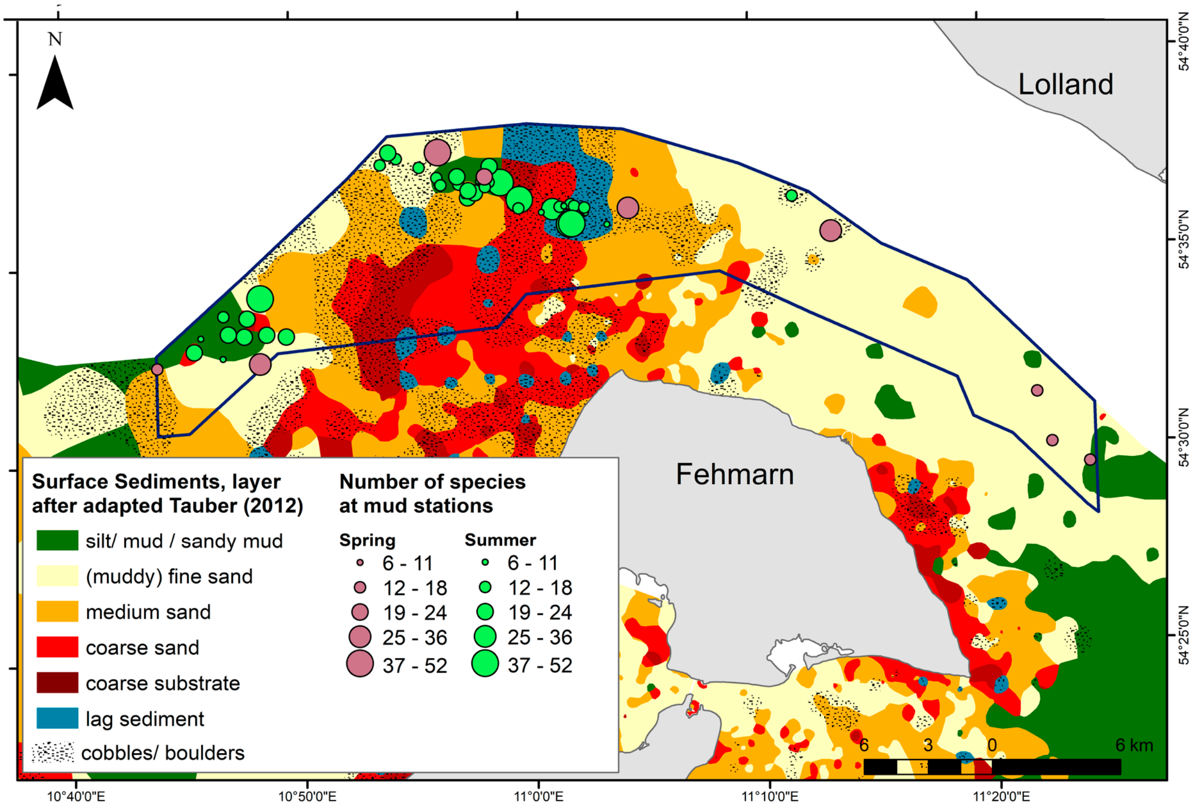

Figure S10. Positioning of stations sampeld in mud.

MODEL INFO:
Observations: 160
Dependent Variable: Number of species
Type: Generalized linear model
Family: poisson
Link function: log

MODEL FIT:
$\chi^2(17) = 566.75$, $p = 0.00$
Pseudo-R^2 (Cragg-Uhler) = 0.97
Pseudo-R^2 (McFadden) = 0.29
AIC = 1448.04, BIC = 1503.39

Standard errors: MLE

	Est.	S.E.	z val.	p
(Intercept)	263.26	48.46	5.43	0.00
Factor (GeoClass) – low	-0.37	0.05	-8.19	0.00
Factor (GeoClass) – medium	-0.06	0.04	-1.40	0.16
Factor (GeoClass) – none	-0.63	0.09	-7.41	0.00
Median grain size	-0.00	0.00	-2.25	0.02
Depth	-0.02	0.00	-5.09	0.00
Salinity	0.03	0.01	5.27	0.00
Year	-0.13	0.02	-5.36	0.00
Factor (substrate) – fine	-95.11	80.60	-1.18	0.24
Factor (substrate) – medium	-290.40	59.58	-4.87	0.00
Factor (substrate) – mud	-317.80	75.45	-4.21	0.00
Season ummer	0.24	0.09	2.78	0.01
Year:substrate – fine	0.05	0.04	1.18	0.24
Year:substrate – medium	0.14	0.03	4.87	0.00
Year:substrate – mud	0.16	0.04	4.21	0.00
substrate fine:Season Summer	-0.20	0.13	-1.50	0.13
substrate medium:Season Summer	-0.09	0.11	-0.83	0.41
substrate mud:Season Summer	-0.50	0.12	-4.13	0.00

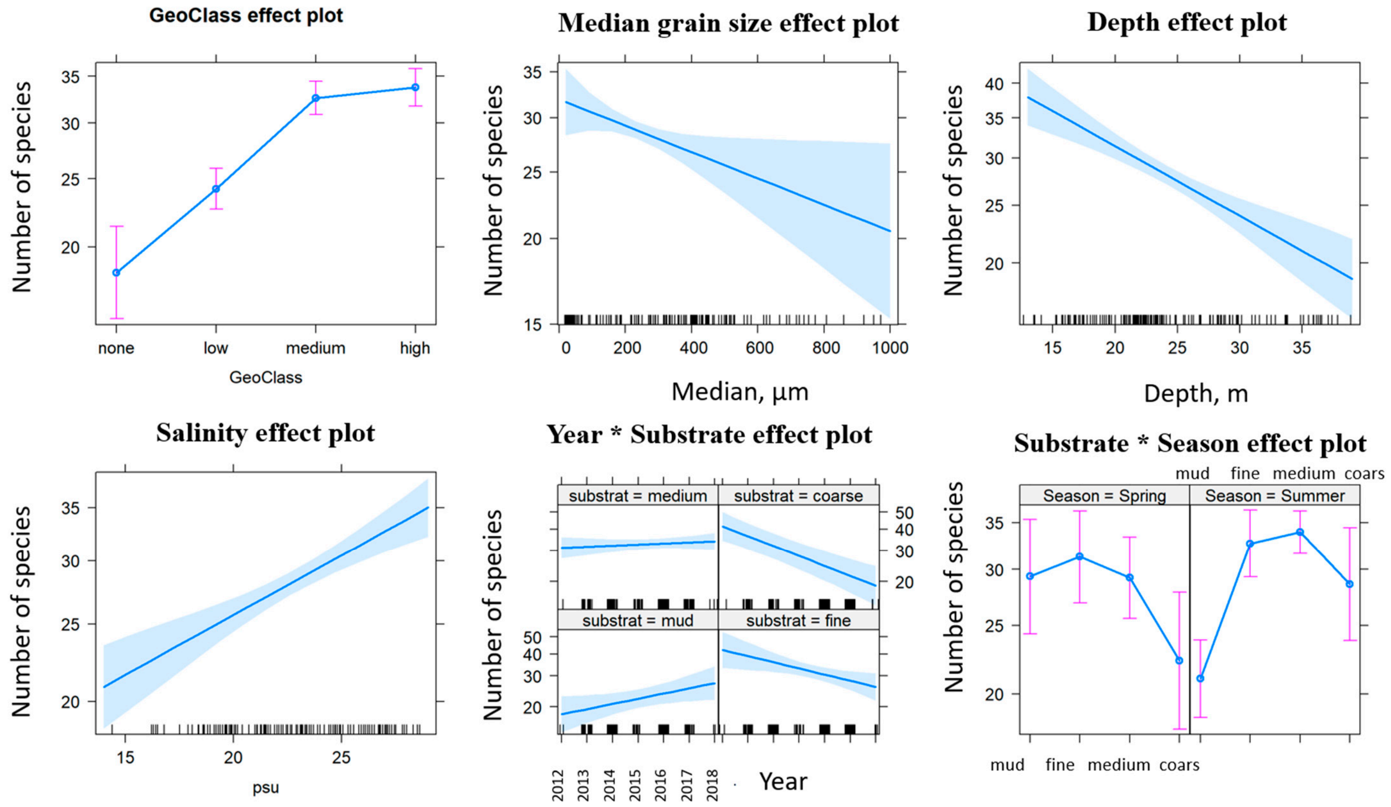

Figure S11. GLM results and effect plots for each predictor in the initial Poisson full model with the two influential points (outliers) based on median grain size in µu units removed from the training dataset.

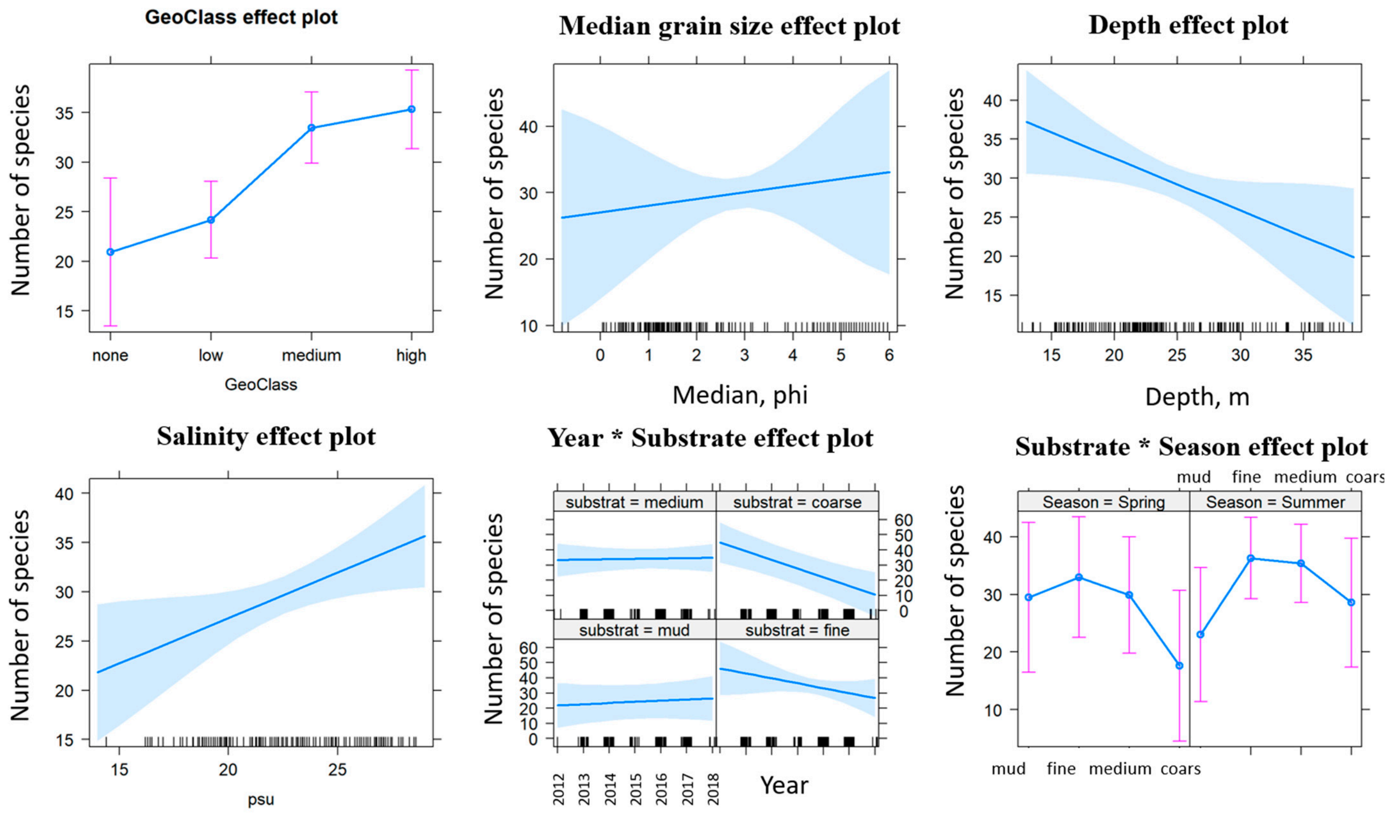

Figure S12. Effect plots for each predictor in the full Poisson model with median grain size transformed in phi units.

Table S6. List of species. The asterisk marks the species classified as "rare" in this study. Of all the 199 species, 84 are shared between all 4 sediment types.

		Sediment Type			
**Phylum**	**Taxa**	**mud**	**fine**	**medium**	**coarse**
Amphipoda	*Ampelisca brevicornis**			x	
	*Ampithoe rubricata**				x
	*Apherusa bispinosa**			x	x
	Autonoe longipes	x		x	x
	Bathyporeia guilliamsoniana			x	x
	*Bathyporeia pilosa**		x	x	
	Caprella linearis	x	x	x	x
	Cheirocratus sundevalli		x	x	x
	Crassicorophium crassicorne	x	x	x	x
	Dexamine spinosa				x
	Dyopedos monacanthus	x	x	x	x
	Dyopedos porrectus	x	x	x	
	Gammarellus homari			x	x
	*Gammarus salinus**			x	
	Ischyrocerus anguipes	x	x	x	
	Medicorophium affine		x	x	
	Megamphopus cornutus	x		x	x
	*Microdeutopus anomalus**			x	
	Microdeutopus gryllotalpa		x	x	x
	Monocorophium insidiosum		x	x	x
	Pariambus typicus			x	
	Phoxocephalus holbolli	x	x	x	x
	Phtisica marina	x	x	x	x
	Protomedeia fasciata	x	x	x	x
Anthozoa	*Actinia equina**				x

	Edwardsia	x	x	x	x
	Halcampa duodecimcirrata	x	x	x	x
	Metridium senile	x	x	x	x
	Sagartia	x	x	x	x
	Urticina felina		x		x
Ascidiacea	*Ciona intestinalis*	x			x
	Dendrodoa grossularia	x	x	x	x
	Molgula manhattensis	x	x	x	x
Bivalvia	*Abra alba*	x	x	x	x
	Arctica islandica	x	x	x	x
	Astarte borealis	x	x	x	x
	Astarte elliptica	x	x	x	x
	Astarte montagui	x	x	x	x
	Corbula gibba	x	x	x	x
	*Crenella decussata**			x	x
	*Ennucula tenuis**	x			
	Hiatella arctica	x	x	x	x
	Kurtiella bidentata	x	x	x	x
	Macoma balthica	x	x	x	x
	Macoma calcarea	x	x	x	
	*Modiolus modiolus**			x	
	Musculus discors			x	x
	Musculus niger	x	x	x	x
	Musculus subpictus	x	x	x	x
	Mya arenaria	x	x	x	
	Mya truncata		x	x	x
	Mytilus edulis	x	x	x	x
	Nucula nitidosa	x			
	Parvicardium pinnulatum	x	x	x	x
	Parvicardium scabrum	x	x	x	x
	Phaxas pellucidus	x	x	x	
	Spisula subtruncata	x	x	x	
	Thracia phaseolina		x	x	
	*Thyasira flexuosa**	x			
Cirripedia	*Balanus crenatus*	x	x	x	x
Cumacea	*Diastylis rathkei*	x	x	x	x
	Eudorella truncatula	x	x	x	x
	Eudorellopsis deformis	x	x	x	
	*Lamprops fasciatus**			x	
Decapoda	*Carcinus maenas**				x
	*Crangon allmanni**	x			
	Crangon crangon	x	x	x	
	*Pagurus bernhardus**		x	x	
Diptera	*Chironomidae*		x	x	
Echinodermata	*Asterias rubens*	x	x	x	x
	Echinocyamus pusillus	x	x	x	x
	Ophiura albida	x	x	x	x
	Psammechinus miliaris	x	x	x	x
Gastropoda	*Acanthodoris pilosa**		x		x
	Amauropsis islandica			x	x
	Bittium reticulatum			x	x
	Boreotrophon truncatus	x	x	x	x

	Brachystomia scalaris	x	x	x	
	Buccinum undatum	x	x	x	x
	*Cadlina laevis**				x
	Diaphana minuta	x	x	x	
	*Ecrobia ventrosa**				x
	*Facelina bostoniensis**	x	x	x	x
	*Hermania scabra**	x			
	*Lacuna pallidula**			x	x
	*Lacuna parva**				x
	Littorina littorea				x
	*Marshallora adversa**				x
	Monophorus perversus				x
	Neptunea antiqua	x	x	x	
	Onchidoris muricata	x		x	x
	Onoba semicostata	x	x	x	x
	Parthenina interstincta		x		
	Peringia ulvae	x	x	x	x
	Philine aperta		x	x	
	Pusillina inconspicua	x		x	
	Retusa obtusa	x	x	x	
	Retusa truncatula	x	x	x	x
	*Tritia reticulata**	x			
	*Vitreolina philippi**			x	
Isopoda	*Pleurogonium rubicundum*	x	x	x	
Mysida	*Gastrosaccus spinifer*	x	x	x	x
	*Praunus flexuosus**			x	
Nemertea	*Cyanophthalma obscura**			x	x
	Lineus ruber	x	x	x	x
	Malacobdella grossa	x	x	x	x
	Nemertea	x	x	x	x
	Tubulanus polymorphus	x	x	x	x
Oligochaeta	*Tubificoides benedii*	x	x	x	x
Phoronida	*Phoronis*	x	x	x	x
Platyhelminthes	*Turbellaria*	x	x	x	x
Polychaeta	*Alitta succinea*			x	x
	*Alitta virens**		x	x	
	Ampharete acutifrons	x	x	x	
	Ampharete baltica	x	x	x	x
	Arenicola marina	x	x	x	x
	Aricidea minuta	x	x	x	x
	Aricidea suecica	x	x	x	x
	*Artacama proboscidea**	x			
	Bylgides sarsi	x	x	x	x
	Capitella capitata	x		x	
	Chaetozone setosa	x	x	x	x
	Dipolydora caulleryi	x	x	x	
	Dipolydora coeca		x	x	
	Dipolydora quadrilobata	x	x	x	x
	*Dodecaceria concharum**	x			
	Enipo kinbergi	x	x	x	
	Eteone barbata	x	x	x	x
	Eteone longa	x	x	x	x

Euchone papillosa	x	x	x	
*Eulalia bilineata**	x			x
Eumida sanguinea		x	x	x
Exogone naidina		x	x	x
Fabriciola baltica	x	x	x	x
Flabelligera affinis		x		
Galathowenia oculata	x	x	x	
Harmothoe imbricata	x	x	x	x
Harmothoe impar	x	x	x	x
Heteromastus filiformis	x	x	x	x
Lagis koreni	x	x	x	x
*Lanice conchilega**			x	x
Laonome kroyeri	x	x	x	x
Lepidonotus squamatus	x	x	x	x
Levinsenia gracilis	x	x	x	x
Lysilla loveni	x	x	x	
*Myrianida**			x	
Neoamphitrite figulus	x	x	x	
Nephtys caeca	x	x	x	x
Nephtys ciliata	x	x	x	x
Nephtys hombergii	x	x	x	x
Nephtys pente	x	x	x	x
Nereimyra punctata	x	x	x	x
*Nereis pelagica**				x
Nicolea zostericola		x	x	x
Nicomache minor	x	x	x	x
Ophelia rathkei			x	x
Paradoneis eliasoni	x	x	x	x
Paraonis fulgens	x	x	x	
*Parexogone hebes**			x	x
Pherusa plumosa	x	x	x	x
Pholoe assimilis	x	x	x	x
Pholoe baltica	x	x	x	x
Phyllodoce groenlandica	x	x	x	
Phyllodoce maculata	x		x	
Phyllodoce mucosa	x	x	x	x
*Poecilochaetus serpens**			x	
Polycirrus medusa	x	x	x	x
Polydora ciliata	x	x	x	
Prionospio steenstrupi	x	x	x	x
Pseudopolydora pulchra	x	x	x	x
Pygospio elegans		x	x	x
Rhodine loveni	x	x	x	
Scalibregma inflatum	x	x	x	x
Scolelepis foliosa		x	x	x
Scoloplos armiger	x	x	x	x
*Sphaerodoropsis baltica**			x	
*Sphaerosyllis hystrix**	x		x	
Spio arndti	x	x	x	x
Spio goniocephala	x	x	x	x
*Spio martinensis**	x			x
*Spiophanes bombyx**			x	
*Spirorbis corallinae**				x

	Spirorbis spirorbis			x	x
	Streptosyllis websteri	x		x	x
	Terebellides stroemii	x	x	x	x
	Tharyx killariensis	x	x	x	
	Tharyx killariensis		x	x	
	Travisia forbesii		x	x	x
	Trochochaeta multisetosa	x	x	x	
Polyplacophora	*Lepidochitona cinerea*	x	x	x	x
Priapulida	*Halicryptus spinulosus**	x			
	Priapulus caudatus	x		x	
Pycnogonida	*Anoplodactylus petiolatus**				x
	Callipallene brevirostris				x
	Nymphon brevirostre	x	x	x	
Sipuncula	*Golfingia**			x	
Tanaidacea	*Heterotanais oerstedii**				x
	*Sinelobus stanfordi**			x	
	Tanaissus lilljeborgi			x	x
	N unique for sediment type	9	2	14	15
